# Destabilization of β-catenin and RAS by targeting the Wnt/β-catenin pathway as a potential treatment for triple-negative breast cancer

**DOI:** 10.1038/s12276-020-0440-y

**Published:** 2020-05-26

**Authors:** Won-Ji Ryu, Jeong Dong Lee, Jong-Chan Park, Pu-Hyeon Cha, Yong-Hee Cho, Jee Ye Kim, Joo Hyuk Sohn, Soonmyung Paik, Kang-Yell Choi

**Affiliations:** 1 Department of Biotechnology, College of Life Science and Biotechnology, Yonsei University, Seoul, 03722 Korea; 2 Department of Human Biology and Genomics, Brain Korea 21 PLUS Project for Medical Sciences, Yonsei University College of Medicine, Seoul, 03722 Korea; 3 Department of Surgery, Yonsei University College of Medicine, Seoul, 03722 Korea; 4 Division of Medical Oncology, Department of Internal Medicine Yonsei University College of Medicine, Seoul, 03722 Korea; 5 Severance Biomedical Research Institute and Department of Medical Oncology, Yonsei University College of Medicine, Seoul, 03722 Korea; 6 CK Biotechnology Inc, Seoul, 03722 Korea

**Keywords:** Targeted therapies, Breast cancer

## Abstract

Triple-negative breast cancer (TNBC) is a severe and heterogeneous disease that lacks an approved targeted therapy and has a poor clinical outcome to chemotherapy. Although the RAS-ERK signaling axis is rarely mutated in TNBC, ~50% of TNBCs show an increased copy number and overexpression of epidermal growth factor receptor (EGFR). However, EGFR-targeted therapies have offered no improvement in patient survival, underscoring the need to explore downstream targets, including RAS. We found that both β-catenin and RAS, as well as epidermal growth factor receptor (EGFR), are overexpressed and correlated with one another in tumor tissues of TNBC patients. KYA1797K, an Axin-binding small molecule reducing β-catenin and RAS expression via degradation and suppressing EGFR expression via transcriptional repression, inhibited the proliferation and the metastatic capability of stable cell lines as well as patient-derived cells (PDCs) established from TNBC patient tissues. KYA1797K also suppressed the stemness of 3D-cultured PDCs and xenografted tumors established by using residual tumors from TNBC patients and those established by the TNBC cell line. Targeting both the Wnt/β-catenin and RAS-ERK pathways via small molecules simultaneously reducing the levels of β-catenin, RAS, and EGFR could be a potential therapeutic approach for TNBC.

## Breast cancer: Dual pathway blockade shrinks aggressive tumors

The triple-negative breast cancer (TNBC), a highly aggressive form of breast cancer may be susceptible to drug therapy that targets two critical signaling pathways, one that governs cell fate and the other that controls the cell cycle. Researchers from Yonsei University in Seoul, South Korea, had previously synthesized a small-molecule called KYA1797K and found that could lower levels of the proteins, β-catenin (a master regulator of cell fate decisions) and RAS (which controls cell division), in colorectal cancer cells. Now, Kang-Yell Choi, Soonmyung Paik, and colleagues have shown that KYA1797K has the same effects on β-catenin and RAS levels in cells taken from patients with TNBC. In cell lines and mouse models, KY1797K suppressed the proliferation and metastatic capability of TNBC, highlighting the therapeutic potential of this strategy degrading both β-catenin and RAS.

## Introduction

Triple-negative breast cancer (TNBC) is a breast cancer subtype that lacks expression of estrogen receptor (ER), progesterone receptor, and human epidermal growth factor receptor 2 (HER2), with a diagnosis rate of 15–20% in breast cancer patients^1–3^. Without these receptors, neoadjuvant chemotherapy is the standard treatment for TNBC. Although ~30% of TNBC patients undergo pathological complete response (pCR) after chemotherapy with excellent survival, those without pCR suffer a grave clinical outcome^4,5^. Despite the recent success of postneoadjuvant capecitabine or immune checkpoint inhibitors in improving the clinical outcome of patients without pCR, there is still a critical need for further reduction in the recurrence rate of these high-risk tumors.

One of the potential treatment targets in TNBC is the EGFR signaling axis^6–8^. Approximately 50% of TNBCs show an increased EGFR copy number, which is associated with poor clinical outcome^9^. However, many clinical trials of drugs targeting EGFR, such as cetuximab, a recombinant EGFR monoclonal antibody, did not yield successful outcomes^10,11^.

The RAS-ERK (extracellular signal regulated kinase) pathway is activated in TNBC patient tissues, which is not attributed to mutations of the genes in this pathway, but to EGFR overexpression^12,13^. EGFR overexpression can occur through increases in gene copy number^9^ or transcriptional induction via the Wnt/β-catenin pathway^14^. The Wnt/β-catenin pathway is active in TNBC patient tissues, and is correlated with tumorigenesis, metastasis, cancer stemness, and poor prognosis in TNBC patients^15,16^. Residual tumors after neoadjuvant chemotherapy were found to have an increased proportion of cancer stem cells (CSCs), suggesting the involvement of CSCs in chemotherapy resistance^17,18^. Therefore, targeting both β-catenin and RAS, as well as upstream EGFR, could be an ideal approach for the treatment of TNBC.

In this study, we identified that the expression levels of β-catenin and RAS were highly increased alongside accumulated EGFR in patient tumor tissues, and their expression levels were correlated in these samples. These results led us to test the efficacy of KYA1797K, a small molecule that simultaneously degrades both β-catenin and RAS proteins via Axin–RGS domain binding^19^, in TNBC both in vitro and in vivo. Due to the major problem of EGFR overexpression in TNBC, the use of KYA1797K, which represses *EGFR* transcription by β-catenin degradation, provides further advantages.

KYA1797K dose-dependently inhibited the growth and transforming capabilities of various TNBC cell lines and primary patient-derived cells (PDCs) with reductions in β-catenin, pan-RAS, and EGFR levels. In addition, KYA1797K further suppressed the invasive characteristics of migratory TNBC cells, which provided support for the potential effectiveness of KYA1797K in preventing metastasis. The inhibition of stem cell characteristics by KYA1797K was also indicated by growth suppression of tumor organoids, with reductions in the cancer stem cell (CSC) markers CD44 and aldehyde dehydrogenase 1 (ALDH1) A3^20,21^.

Additional support for KYA1797K in TNBC suppression was indicated by the growth reduction of tumors generated by MDA-MB-468 TNBC cells or the residual tumor tissues of TNBC patients treated with neoadjuvant chemotherapy. We also confirmed the inhibitory effects of KYA1797K on TNBC patient-derived xenograft (PDX) tumors; these effects occurred through the modulation of β-catenin, RAS, and EGFR expression. Collectively, the small molecule induced destabilization of β-catenin and RAS, which leads to inhibition of their respective pathways and to inhibition of EGFR expression; this could provide insight into a potential therapy for TNBC patients.

## Materials and methods

### Tissue microarray (TMA)

TMAs for normal-adjustment breast and TNBC tissues (BC081120b) were purchased from US Biomax (Rockville). Immunohistochemistry was performed with β-catenin, pan-RAS, or EGFR antibodies. Bright-field microscopy (Nikon; Melville, New York; ECLIPSE 80i) was used to obtain images of each specimen. For quantification of the expression levels of nuclear or cytoplasmic proteins, the TMA images were quantified using the IHC profiler plugin for NIH Image software^22^.

### Patient-derived xenograft (PDX) and cell line xenograft experiments

PDX mice were established from the residual tumor tissues of two TNBC patients after neoadjuvant chemotherapy, as described previously^23^. All studies were approved by the Institutional Review Board of Severance Hospital, Seoul, South Korea (4-2012-0705). Patient tumor samples were collected from patients in accordance with the relevant IRB guidelines. Briefly, 5-week-old female Balb/c nude mice (Charles River, Japan) or female NOG mice (NOD/Shi-scid, IL-2 Rγ null; CIEA, Japan) were purchased and acclimatized for 1 week, and used for the generation of xenograft mice with MDA-MB-468 cell lines or patient tumor tissues.

The Balb/c nude mice were injected subcutaneously in the dorsal flank with 5 × 10^6^ MDA-MB-468 cells in 200 µl 2:1 PBS:Matrigel (BD Biosciences, San Jose, CA). Patient tumors were sliced into 3 × 3 × 3 mm^3^ fragments, and then subcutaneously implanted into the flanks of NOG mice. Drug treatment was initiated when the mean tumor volume reached between 150 and 200 mm^3^. Mice were randomly assigned to specific treatment groups. KYA1797K was injected intraperitoneally at a dose of 25 mg/ml daily. The sizes of the implanted tumors were measured 2–3 times a week using Vernier calipers, and the tumor volume was calculated as follows: (length × width^2^)/2. Mice were sacrificed, and the tumors were isolated, weighed, sliced, and fixed in formalin or liquid nitrogen for further analyses.

### Patient-derived cells (PDCs)

PDCs were established from residual tumor tissue from primary TNBC after neoadjuvant chemotherapy; the protocol for PDC establishment was as described by Liu et al.^24^. Epithelial cells were cocultivated with irradiated (3000 rad) Swiss 3T3 fibroblasts (J2 strain) in F medium [3:1 (v/v) F12 nutrient Mixture (Ham)–Dulbecco’s modified Eagle’s medium (Invitrogen, Waltham, MA, USA), 5% fetal bovine serum (FBS; Gibco; Gaithersburg, MD), 0.4 µg/ml hydrocortisone (Sigma-Aldrich, St. Louis, MO), 5 µg/ml insulin (Sigma-Aldrich), 8.4 ng/ml cholera toxin (Sigma-Aldrich), 10 ng/ml epidermal growth factor (EGF; Invitrogen), and 24 µg/ml adenine (Sigma-Aldrich)] with the addition of 5 to 10 µM/L Y-27632 (Enzo Life Sciences, Seoul, South Korea).

### Cell culture and drug treatment

Human TNBC stable cell lines (MDA-MB-436, MDA-MB-468, and 4T1) were obtained from the American Type Culture Collection (ATCC; Manassas, Virginia). BT549 cells were provided by S.-J. Lee (Hanyang University, Korea). Normal-like breast cells, MCF10A, were provided by D.S. Min (Pusan University, Korea). Cells were cultured in DMEM or RPMI (Gibco) containing 10% FBS, 100 U/ml penicillin, 100 µg/ml streptomycin (Gibco), and 5% CO_2_ at 37 °C. All chemicals were dissolved in dimethyl sulfoxide (DMSO; Sigma-Aldrich) for the in vitro studies. Unless otherwise indicated, KYA1797K was used at a concentration of 25 µM for 24 h.

### Three-dimensional TNBC primary tumor cultures

The detailed protocol and reagents for primary tumor organoids were as described by DeRose et al.^25^. Briefly, primary TNBC patient cells were suspended in modified M87 medium, mixed with Matrigel (BD Biosciences), and plated in 48-well plates. After Matrigel polymerization, modified M87 medium [advanced DMEM/F12 supplemented with FBS (Gibco), insulin-transferrin-selenium-X supplement (×100) (Invitrogen), penicillin-streptomycin-glutamine liquid (×100) (Invitrogen), hydrocortisone (Sigma-Aldrich), cholera toxin (Sigma-Aldrich), 3,3′,5-triiodo-L-thyronine (T3) (Sigma-Aldrich), β-estradiol (E2) (Sigma-Aldrich), (±)-isoproterenol hydrochloride (Sigma-Aldrich), ethanolamine (Sigma-Aldrich), and O-phosphorylethanolamine (Sigma-Aldrich) containing growth factors (50 ng/ml EGF, Peprotech, Rocky Hill, NJ 08553)] was overlain. On the first day after seeding, the medium was also supplemented with 10 mM ROCK inhibitor Y-27632 (Sigma-Aldrich) to avoid anoikis. Fresh media with growth factors was changed every 2 days for maintenance. For treatment of tumor organoids with KYA1797K, media with KYA1797K or DMSO was changed every 2 days from the second day after seeding.

### Wound-healing assay

TNBC cells were seeded in six-well plates coated with collagen (500 μg/ml). After reaching confluence, cells were scratched with a 200 µl tip, and culture media was exchanged with 10% RPMI containing KYA1797K or DMSO. Migrated cells were quantified using NIS-Elements AR 3.1 software (Nikon). Screenshots were captured from video files and were represented as images at several time points. Mean ± SD are reported based on three or five biological replicates.

### Transwell or invasion assay

PDCs or TNBC cell lines were seeded at a density of 3 × 10^5^ onto noncoated or Matrigel-coated chambers (BD Bioscience) with KYA1797K or DMSO. Cells were allowed to invade for 24 h. After clearing cells on the inner surface of the chamber, the cells on the outer surface were fixed in 4% paraformaldehyde (PFA) for 15 min and stained with crystal violet for 20 min. The chambers were dipped in distilled water to remove excess staining and allowed to dry. Representative images were captured by microscopy (TE-2000U, Nikon). Mean ± SD are reported based on three biological replicates.

### Spheroid culture

BT549 or 4T1 cells were seeded in 90-mm Petri dishes at 1 × 10^4^ cells/well. CSC media was supplemented with DMEM/F12 (Invitrogen) containing human recombinant EGF (20 ng/ml, Invitrogen) and human recombinant basic fibroblast growth factor (bFGF, 20 ng/ml, Invitrogen). After 24 h, spheroids were treated with KYA1797K or DMSO for 7 days, with whole media changed every 2 days. Dead cells were removed by centrifugation, and the remaining spheroids were washed with cold PBS and fixed with acetone for 24–48 h.

### Immunoblotting analysis

Cells were washed in ice-cold PBS and lysed using radioimmunoprecipitation assay (RIPA) buffer containing 20 mM NaF, 1 mM sodium vanadate, 10 mM Tris-HCl at pH 7.5, 5 mM EDTA, 150 mM NaCl, 1% NP-40, 1 mM PMSF, and protease inhibitor cocktail (Millipore; Billerica, MA, USA). Tissues were homogenized and dissolved in RIPA buffer. Proteins were separated by a 10–12% sodium dodecyl sulfate-polyacrylamide gel (SDS-PAGE) and transferred to a nitrocellulose membrane (GE Healthcare Life Sciences, Pittsburgh, PA, USA). Immunoblotting was performed with the following primary antibodies: anti-pan-RAS monoclonal (clone Ras10; Millipore; MABS195; 1:3000), anti-β-catenin (Santa Cruz, Dallas, TX, USA; sc-7199; 1:3000), anti-p-ERK (Cell Signaling Technology, Beverly, MA, USA; #9101S; 1:1000), anti-ERK (Santa Cruz; sc-514302; 1:5000), anti-p-Akt (Cell Signaling Technology; #4060S; 1:1000), anti-EGFR (abcam; Cambridge, USA; ab131498; 1:1000), anti-N-cadherin (BD Biosciences; 1:1000), anti-α-SMA (abcam; ab5694; 1:1000), anti-Vimentin (Abcam; ab92547; 1:2000) and anti-β-actin (Santa Cruz; sc-47778; 1:5000). Horseradish peroxidase-conjugated anti-mouse (Cell Signaling Technology; #7076; 1:3000) or anti-rabbit (Bio-Rad, Hercules, USA; 1:3000) secondary antibodies were used. Bands were detected by enhanced chemiluminescence (Amersham Biosciences, Issaquah, WA, USA) using a luminescent image analyzer (LAS-3000; Fuji Film, Tokyo, Japan).

### Immunohistochemistry

For immunohistochemistry (IHC) staining, 4-µm paraffin-embedded tissue sections were treated with citrate buffer (pH 6.0) and autoclaved for 15 min. The sections were then blocked with 5% bovine serum albumin (BSA) and 1% normal goat serum (NGS; Vector Laboratories, Burlingame, CA, USA) in PBS for 1 h for human and mouse tumor samples. For fluorescent IHC, sections were incubated with primary antibody [anti-β-catenin (BD Biosciences; #610154; 1:200), anti-pan-RAS monoclonal (1:100), anti-EGFR (1:100), or anti-CD44 (ProteinTech; Rosemont, IL; 1:100)] overnight at 4 °C, followed by incubation with anti-mouse Alexa Fluor 488 (Life Technologies, Camarillo, CA; A11008; 1:500) or anti-rabbit Alex Fluor 555 (Life Technologies; A21428; 1:500) secondary antibodies for 1 h at room temperature. Sections were counterstained with 4,6-diamidino-2-phenylindole (DAPI; Sigma-Aldrich) and mounted in gel/mount media (Biomeda Corporation, Foster City, CA, USA). All processes were conducted in the dark, humid chambers. A confocal microscope (LSM510; Carl Zeiss) was used to visualize the fluorescence signal at excitation wavelengths of 488 nm (Alexa Fluor 488), 543 nm (Alexa Fluor 555), or 405 nm (DAPI). At least three fields per section were analyzed. For peroxidase IHC analysis, 3.45% H_2_O_2_ (Samchun Chemicals, Seoul, South Korea) was applied to sections to block endogenous peroxidase activity for 15 min. Before incubating sections with mouse primary antibody, mouse IgG was blocked using the mouse-on-mouse (M.O.M.) IgG blocking kit (Vector Laboratories). Sections were incubated with anti-β-catenin, anti-pan-RAS, or anti-EGFR primary antibodies overnight at 4 °C, followed by incubation with biotinylated anti-mouse (Dako, Santa Clara, CA; E-0433; 1:300) or biotinylated anti-rabbit (Dako, E-0353; 1:300) secondary antibodies for 1 h at room temperature. The samples were then incubated in avidin biotin complex solutions (Vector Laboratories), stained with 3,3-diaminobenzidine (DAB; Dako) for a maximum of 5 min, and counterstained with Mayer’s hematoxylin (Muto, Bunkyou-ku, Tokyo, Japan). All incubations were performed in humid chambers. Signals were analyzed using a bright-field microscope (TE-2000U; Nikon).

### Immunocytochemistry

Cells grown on gelatin-coated cover glasses were fixed in 4% PFA for 10 min, followed by permeabilization with 0.1% Triton X-100 for 15 min, blocking in 5% BSA for 1 h, and primary antibody incubation overnight at 4 °C. Primary antibodies were removed, and cells were washed with PBS and incubated for 1 h at room temperature with either Alexa Fluor 488- or Alexa Fluor 555-conjugated IgG secondary antibodies (Invitrogen). Cell nuclei were counterstained by incubating the cells in DAPI. Immunofluorescence images were captured using a confocal microscope (LSM510; Carl Zeiss).

### Cell proliferation and colony-formation assays

To assay cell proliferation, TNBC PDCs and stable cell lines (BT549, MDA-MB-436, MDA-MB-468, and 4T1) were plated at a density of 8–10 × 10^3^ cells/well in a 24-well plate. Cells were then treated with 5 or 25 µM KYA1797K or with DMSO for 72 or 120 h. Next, 3-(4,5-dimethylthiazol-2-yl)-2-5-diphenyltetrazolium bromide (MTT; AMRESCO, Solon, OH) reagent was added to each well at a concentration of 0.5 mg/ml. After incubation for 1 h at 37 °C, insoluble purple formazan was obtained by incubating in 500 μl (24-well plate) of DMSO for 20 min. The absorbance of the formazan product was determined at 590 nm every 24 h. For colony-formation assays, cells were seeded in 12-well plates (100–500 cells/well for TNBC cells). DMSO control or KYA1797K was added to the cells with medium changes every 3 days until visible colonies formed. At the end of the experiment, cells were fixed in 4% PFA for 30 min and stained with 0.5% crystal violet in 20% ethanol for 30 min.

### Single-cell migration assay

PDCs were seeded at a density of 1 × 10^4^ cells on gelatin-coated chambers. After 24 h, single cells were imaged using a time-lapse video microscope. The videos and the migratory paths of single cells were constructed using NIS-Elements AR 3.1 (Nikon).

### Reverse transcription and quantitative real-time PCR

CRCs and BT549 cells were seeded at a density of 3 × 10^5^ cells/well in six-well plates and then treated with KYA1797K (25 µM) for 24 h. The cells were washed with PBS, and the total RNA was isolated using TRIzol reagent (Invitrogen) following the manufacturer’s instructions. The total RNA (2 μg) was reverse-transcribed using 200 units of reverse transcriptase (Invitrogen) in a 40 μl reaction performed at 42 °C for 1 h. The resulting cDNA (2 μl) was amplified in a 40 μl reaction mixture containing 10 mM dNTP (Takara; Mountain View, CA), 10 pmol of the primer set (Bioneer), and 1 unit of Taq DNA polymerase (Invitrogen). The following primer sets were used: *CTNNB1* (which encodes β-catenin), forward 5′-ACA AGC CAC AAG ATT ACA AGA A-3′ and reverse 5′-GCA CCA ATA TCA AGT CCA AGA-3′; *H-RAS*, forward 5′-GGA AGC AGG TGG TCA TTG-3′ and reverse 5′-AGA CTT GGT GTT GTT GAT GG-3′; *N-RAS*, forward 5′-AAG AGT TAC GGG ATT CCA TTC-3′ and reverse 5′-CCA TCA TCA CTG CTG TTG A-3′; *K-RAS*, forward 5′-AAA CAG GCT CAG GAC TTA G-3′ and reverse 5′-GTA TAG AAG GCA TCA TCA AC-3′; *EGFR*, forward 5′-ATG CCC GCA TTA GCT CTT AG-3′ and reverse 5′-GCA ACT TCC CAA AAT GTG CC-3′; and β-actin, forward 5′-AGA GCT ACG AGC TGC CTG AC-3′ and reverse 5′-AGC ACT GTG TTG GCG TAC AG-3′.

### Statistical analyses

Statistical analyses were performed using Microsoft Excel or GraphPad Prism 5 software (GraphPad Software). All data are represented as the mean ± SD. For the TMA analysis of normal-adjacent tissue (NAT) and TNBC tumor tissue, statistical analysis was also performed using GraphPad Prism 5 software. Statistically significant differences were determined using Student’s *t* tests, and statistically significant *P*-values are presented as follows: *P < 0.05; **P < 0.005; and ***P < 0.0005.

## Results

### Elevated β-catenin, RAS, and EGFR levels were positively correlated in TNBC patient tissues

To validate the involvement of β-catenin and RAS in the tumorigenesis of TNBC, their expression levels and relationship with EGFR expression in breast cancer patient tissues were investigated by IHC analyses of TMA. Histoscores (H-scores) for nuclear β-catenin, which reflects Wnt/β-catenin pathway activity, alongside pan-RAS and EGFR expression were obtained using analysis with the IHC-Profiler plugin for NIH Image software.

**Fig. 1:**
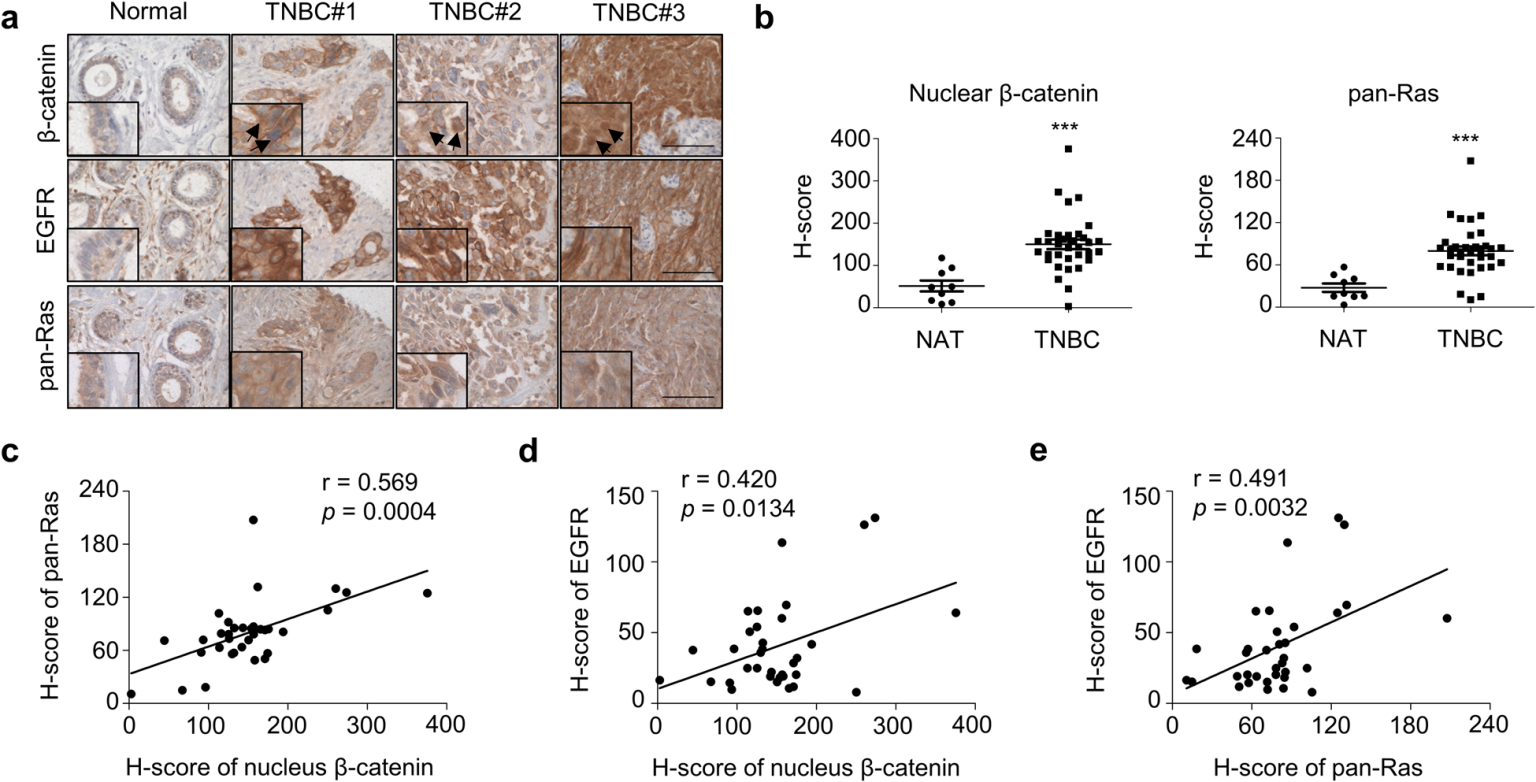
The increased expression and correlation of β-catenin, pan-RAS, and EGFR in the tumor tissues of TNBC patients. **a** IHC analyses of the TMA including TNBC tissues (*n* = 34) and NATs (*n* = 10) were performed using β-catenin, pan-RAS, or EGFR antibodies. Scale bar represents 200 μm. **b** Quantified H-score of nuclear β-catenin (*p* < 0.0005) and cytosolic and membranous pan-RAS (*p* < 0.0005) in NATs and TNBC tissues. (GraphPad Prism 5, *t* test with two-tailed *p*-value) **c** A positive correlation (*p* = 0.0004; Pearson’s correlation coefficient) between the expression of nuclear β-catenin and pan-RAS in TNBC tissues. **d**, **e** A positive correlation (Pearson’s correlation coefficient) between the expression of EGFR with nuclear β-catenin (*p* = 0.0134) or pan-RAS (*p* = 0.0032) in TNBC tissues. *P < 0.05, **P < 0.005, ***P < 0.0005.

The expression levels of nuclear β-catenin and pan-RAS were significantly increased in TNBC patient tissues (*n* = 34) compared with adjacent normal tissues (NAT, *n* = 9) (Fig. 1a, b) and significantly correlated with each other (*p* = 0.0004, *r* = 0.569) (Fig. 1c). To further investigate the potential association of EGFR with the Wnt/β-catenin and RAS/ERK pathways in TNBC, we examined correlation between β-catenin, pan-RAS, and EGFR. EGFR levels were positively correlated with β-catenin (*p* = 0.0134, *r* = 0.420) and pan-RAS (*p* = 0.0032, r = 0.491), respectively (Fig. 1d, e; Supplementary Fig. 1).

Moreover, we also observed a correlated change in Wnt/β-catenin and RAS-ERK/RAS-Akt pathway status in MCF10A cell lines by modification of Wnt/β-catenin signaling. The levels of β-catenin, RAS, and EGFR, as well as the RAS downstream effectors ERK and Akt, were significantly increased after Wnt3a treatment in MCF10A cells (Supplementary Fig. 2).

### KYA1797K downregulates β-catenin, RAS, and EGFR and suppresses the proliferation and colony formation of TNBC cells

**Fig. 2:**
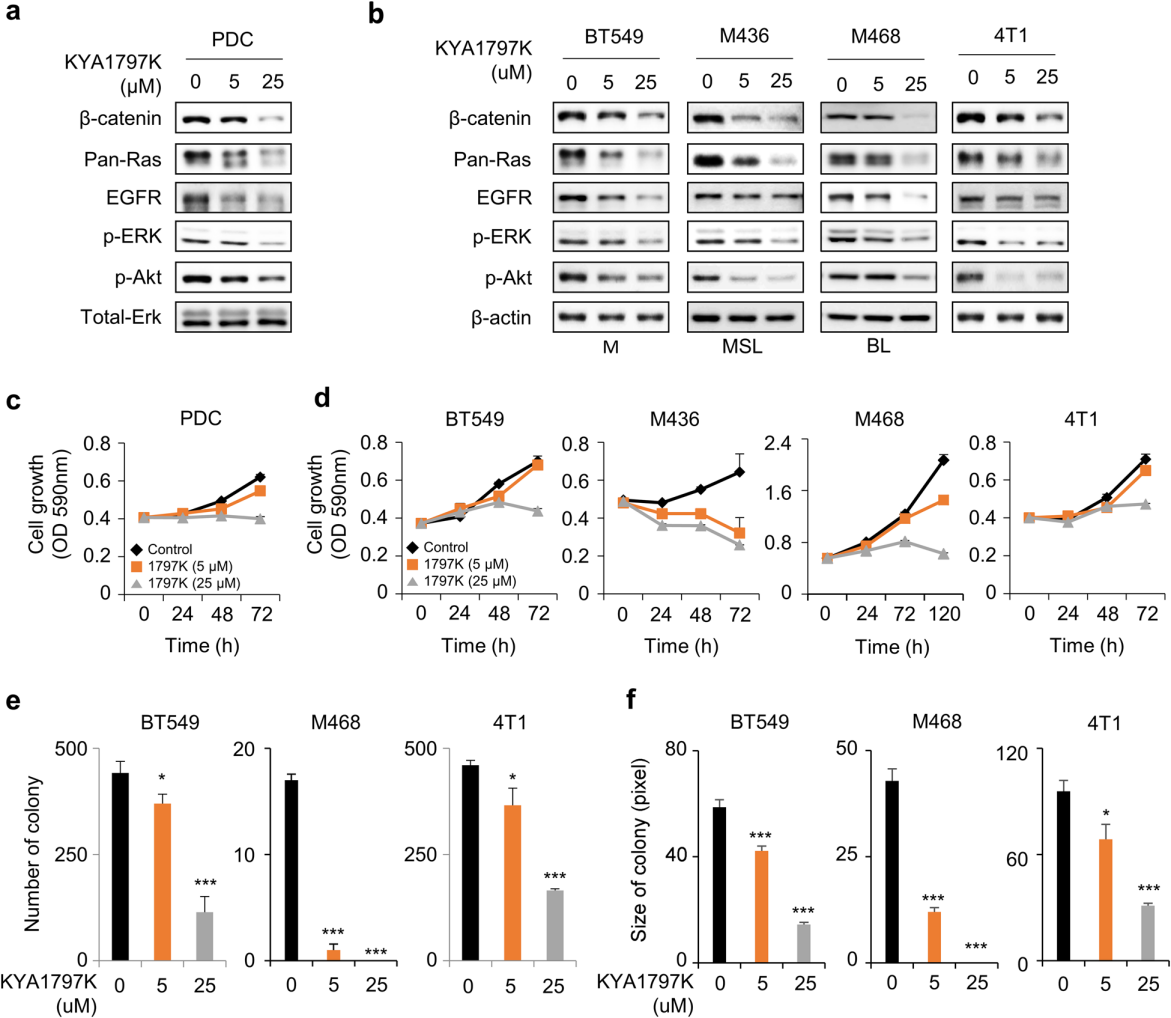
Effects of KYA1797K on TNBC PDCs and cell lines. **a**, **b** TNBC cells were cultured and treated with 5 or 25 µM KYA1797K for 24 h. Immunoblot analyses of whole-cell lysates (WCLs) with the indicated antibodies. **c**, **d** PDCs, human TNBC cell lines, and 4T1 mouse TNBC cells were dose-dependently treated with KYA1797K. Cell proliferation was measured and quantified using MTT assay (*n* = 3). **e**, **f** Colony-formation assay was performed in TNBC cells treated with 5 or 25 µM KYA1797K for 14 days. The colonies in **e** and **f** were photographed and quantified from independent experiments (mean ± SEM; *n* = 3) based on two-sided Student’s *t* test between control and experimental samples, respectively. *P < 0.05, **P < 0.005., ***P < 0.0005. M = mesenchymal-like, MSL = mesenchymal stem-like, BL = basal-like.

Using PDCs established from a residual tumor after neoadjuvant chemotherapy from a TNBC patient, the dose-dependent inhibitory effects of KYA1797K were confirmed by decreasing the levels of β-catenin and pan-RAS with ERK and Akt inactivation in TNBC PDCs (Fig. 2a). A similar reduction was observed in the three human TNBC cell lines and the mouse TNBC cell line 4T1 (Fig. 2b). However, the mRNA levels of *K-, H-, N-RAS*, and *CTNNB1* (which encodes β-catenin) were not affected (Supplementary Fig. 3a). KYA1797K decreased the mRNA level of *EGFR*, a transcriptional target of β-catenin^14^, which resulted in a reduction of EGFR protein level (Fig. 2a, b; Supplementary Fig. 3b). The growth rates of TNBC cells were dose-dependently decreased by KYA1797K treatment (Fig. 2c, d). In addition, the number and size of colonies were also reduced by KYA1797K in TNBC cells (Fig. 2e, f; Supplementary Fig. 4).

### KYA1797K inhibits the migration and invasion of TNBC cells

**Fig. 3:**
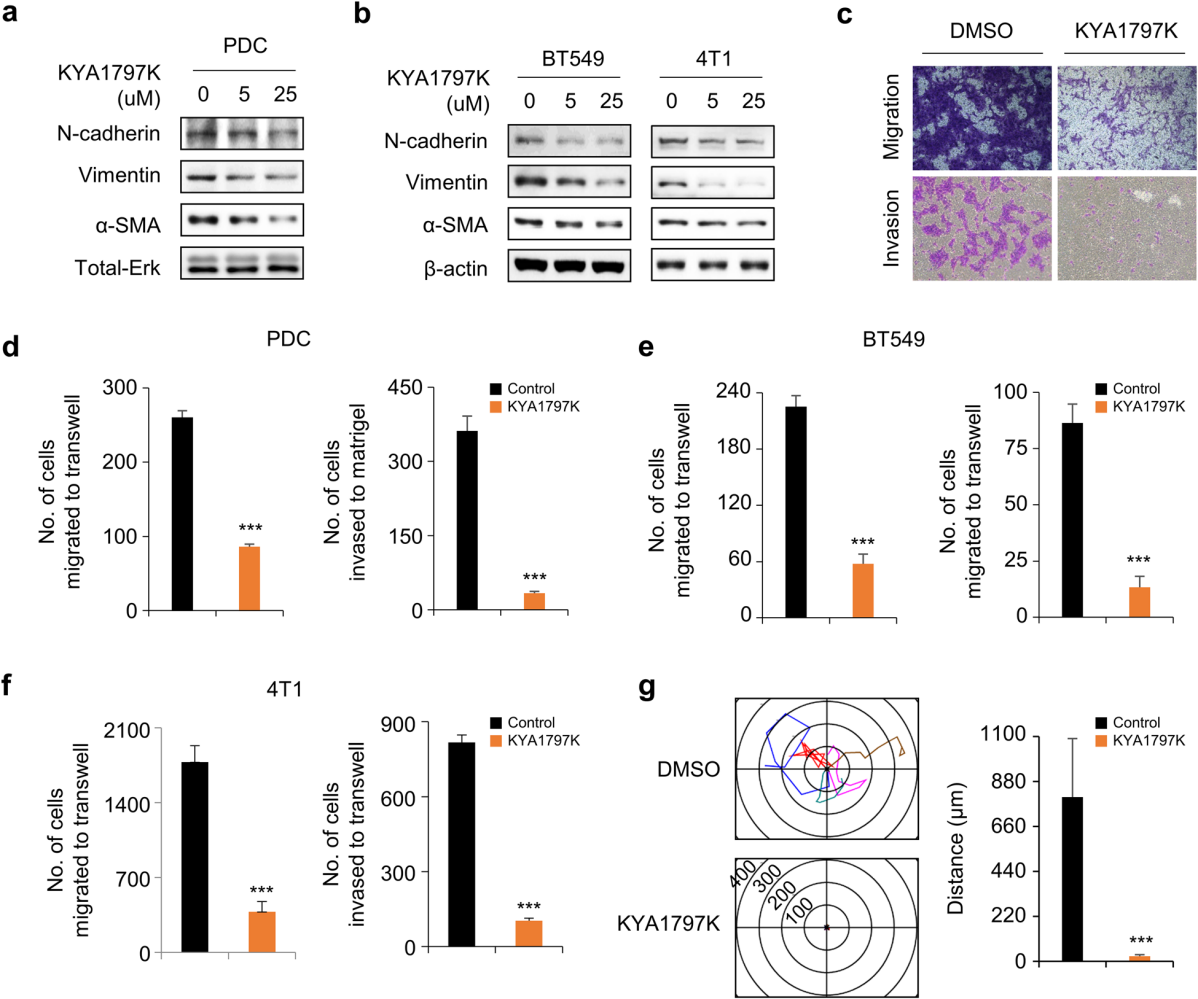
Inhibitory effects of KYA1797K on migration and invasion in TNBC cells. **a**, **b** IB analyses of WCLs from PDCs (**a**) or cell lines (**b**) were performed using indicated antibodies. **c**–**f** Migration or invasion assay were performed using noncoated or Matrigel-coated chambers on PDCs or BT549 and 4T1 cells. Migrated or invaded cells were stained with 0.25% crystal violet. The representative images in **c** were captured with a Nicon TE-2000U. The number of migrated or invaded cells in PDCs (**d**) or BT549 and 4T1 cells (**e**, **f**) were quantified. **g** Single-cell migratory pattern was monitored using real-time imaging microscopy (Eclipse Ti, Nikon) in PDCs (*n* = 5). A collagen coating was applied before seeding the cells. Data values represent the mean ± SEM. *P < 0.05, **P < 0.005, ***P < 0.0005.

To identify the in vitro effects of KYA1797K on the metastatic capacity of TNBC cells, we utilized a Transwell assay. Metastatic markers such as N-cadherin, vimentin, and α-SMA were dose-dependently reduced by KYA1797K in TNBC PDCs and TNBC cell lines (Fig. 3a, b). In accordance with these effects, KYA1797K significantly decreased the number of migrated or invaded cells compared with the control treatment in both TNBC PDCs and cell lines (Fig. 3c–f). Following the wound-healing assay, the number of cells that migrated into the scratch wound area was also significantly reduced in the group treated with KYA1797K compared with the group treated with the control (Supplementary Fig. 5a, b). In addition, we confirmed inhibited cell motility at the single-cell level in TNBC PDC cells treated with KYA1797K (Fig. 3g). Overall, the data showed that the migratory and invasive abilities of TNBC cells can be regulated via suppression of both the Wnt/β-catenin and RAS-ERK pathways.

### KYA1797K suppresses the CSC characteristics of TNBC

**Fig. 4:**
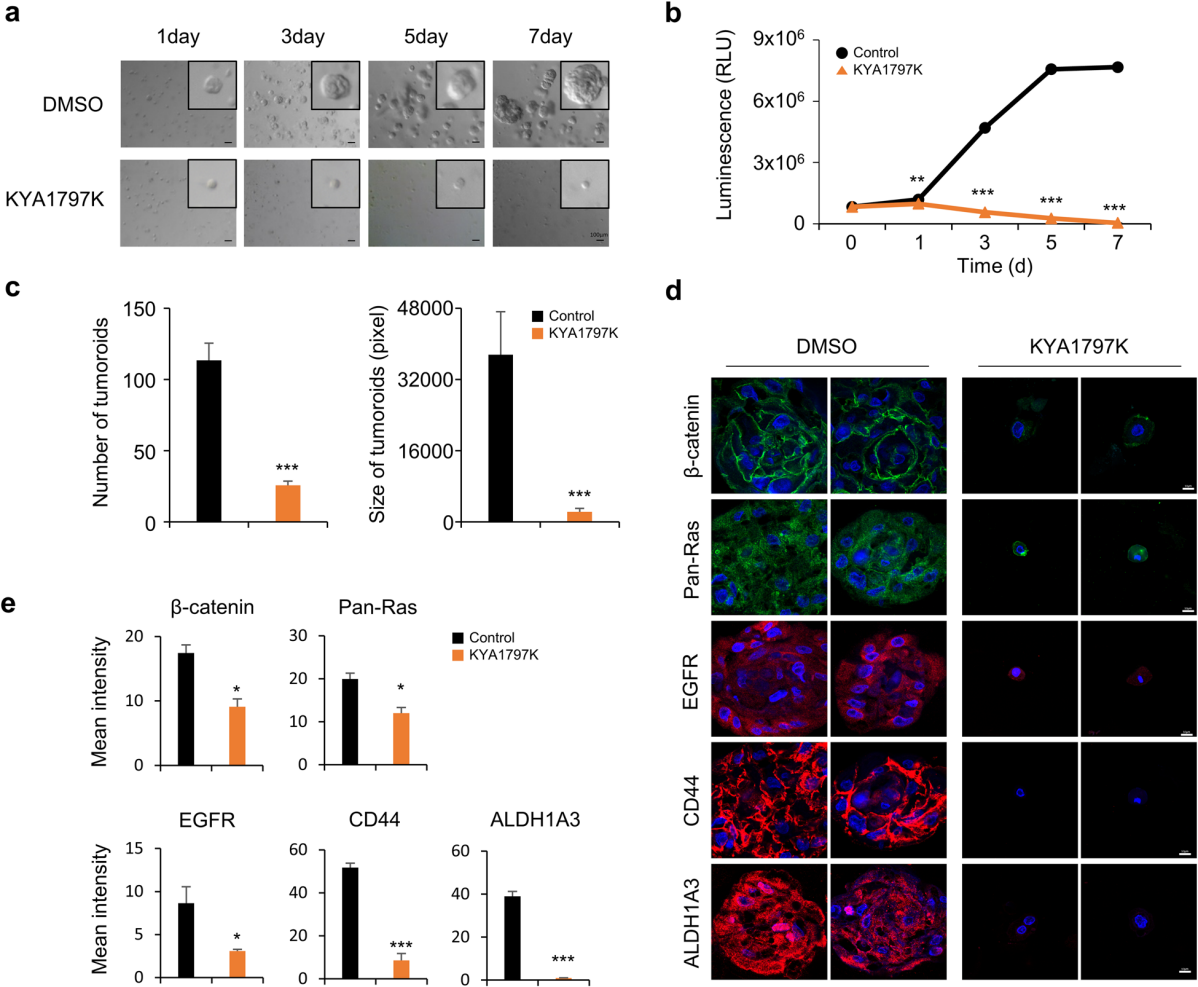
Effects of KYA1797K on cancer stemness of TNBC cells. TNBC PDCs were cultured using a Matrigel-based 3D culture system described in “Materials and methods”. **a** The effect of KYA1797K (25 μM) on tumor organoids of TNBC PDCs was tested for 7 days. Representative images were captured every 2 days. Scale bar = 100 μm. **b** Viable cells were measured using luminescence production (CellTiter-Glo^®^ Luminescent Cell Viability Assay). **c** The sizes and numbers of tumor organoids of TNBC PDCs were measured and quantified using the Image J program. Statistical significance was determined by two-side Student’s *t* test. **d** Immunocytochemistry (ICC) analyses were performed with the indicated antibodies. Images were captured using a Zeiss confocal microscope. Scale bar = 10 μm. **e** Mean intensity of fluorescence for each markers was quantified for at least three different tumor organoids using ZEN microscope software (Carl Zeiss Microscopy). Data values represent the mean ± SEM. *P < 0.05, **P < 0.005, ***P < 0.0005.

Since the Wnt/β-catenin pathway is known to have an important role in stem cell generation and division, we also investigated the effects of KYA1797K on CSCs using a 3D culture system with PDCs established from a residual tumor after neoadjuvant chemotherapy from a TNBC^26^. The growth of tumor organoids was completely suppressed by KYA1797K treatment (Fig. 4a, b), and the number and size of tumor organoids were also significantly decreased (Fig. 4c). KYA1797K also suppressed β-catenin, RAS, and EGFR levels, as shown by IHC analyses (Fig. 4d, e). In addition, we confirmed that the expression levels of CD44 and ALDH1A3, markers of CSCs, in tumor organoids were significantly lowered after KYA1797K treatment. Using a spheroid culture system, we also verified the inhibitory effect of KYA1797K on the stemness of TNBC cell lines; in this model, KYA1797K effectively reduced both the number and size of spheroids derived from BT549 and 4T1 cells (Supplementary Fig. 6a, b).

### KYA1797K suppressed the growth of TNBC PDX tumors

**Fig. 5:**
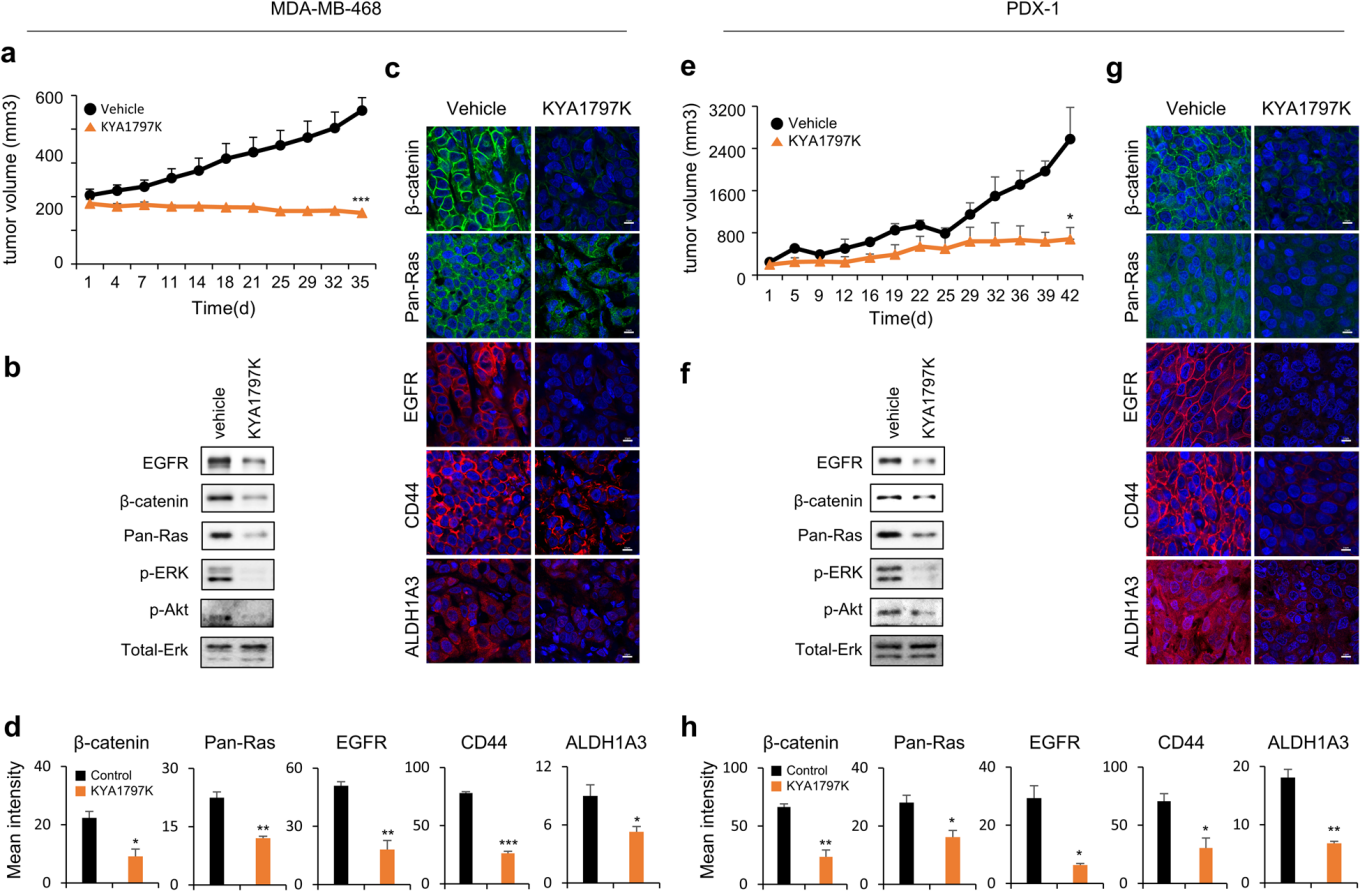
Effects of KYA1797K on the growth of tumors derived from a TNBC cell line xenograft and a patient-derived xenograft (PDX). Mice-bearing TNBC tumors established from MDA-MB-468 cells or patients who showed resistance to adjuvant chemotherapy were treated with vehicle or KYA1797K (25 mg/ml) when the tumor burden reached a volume of 150–200 mm^3^ (MDA-MB-468: vehicle *n* = 3, KYA1797K *n* = 4; PDX-1: vehicle *n* = 3, KYA1797K *n* = 3). KYA1797K was administered as described in “Materials and methods”. Subcutaneous tumor volumes were measured using calipers. **a**, **e** Tumor volumes were measured for 35 days (MDA-MB-468) or 42 days (PDX-1). **b**, **f** IB analyses with the indicated antibodies using WCLs of MDA-MB-468 cells or PDX-1 tumors. IHC analyses were performed on formalin-fixed 4-μm paraffin sections of tumors-derived MDA-MB-468 cells (**c**, **d**) or PDX-1 (**g**, **h**) using the indicated antibodies. Mean fluorescence intensity for each marker was quantified by using a ZEN microscope software (Carl Zeiss Microscopy). Data values represent the mean ± SEM. *P < 0.05, **P < 0.005., ***P < 0.0005.

The in vivo effects of KYA1797K in TNBC were also assessed using a xenograft mouse model that produces tumors after implantation of MDA-MB-468 cells overexpressing EGFR. The growth rates of tumors in mice injected with KYA1797K were significantly slower than those injected with the vehicle (Fig. 5a). In accordance with the suppression of tumor growth, KYA1797K significantly decreased β-catenin, pan-RAS, and EGFR levels as well as ERK and Akt activities, downstream of RAS (Fig. 5b). The reduction in β-catenin, pan-RAS, and EGFR levels was confirmed by IHC analyses. The CSC characteristics of these tumors, indicated by the markers CD44 and ALDH1A3, were also reduced after KYA1797K treatment (Fig. 5c, d).

To further validate the preclinical strategy for degrading β-catenin and RAS in TNBC, we evaluated the effects of KYA1797K on the growth of PDX tumors derived from two TNBC patients. Both PDXs were derived from residual invasive tumor cells after neoadjuvant chemotherapy (Supplementary Table 1).

PDX tumors after three passages in NOG mice were sliced and transplanted into the flanks of NOG mice, which were treated with KYA1797K after tumor sizes reached 150–200 mm^3^. In both PDX models, tumor growth was inhibited along with a reduction in the level of biological targets. The PDX-1 tumor growth rate was significantly lowered by KYA1797K treatment (*p* < 0.01) (Fig. 5e), and the reduction in β-catenin, pan-RAS, and EGFR expression levels in tumor tissues was confirmed (Fig. 5f–h). KYA1797K also reduced CD44 and ALDH1A3 expression levels in PDX-1 tumors (Fig. 5g, h). In addition, ERK and Akt activities, the downstream effectors of RAS, were markedly reduced in PDX-1 tumors in accordance with the inhibition of tumor growth (Fig. 5b). Therefore, KYA1797K suppressed the growth of tumors derived from TNBC cell lines and patient tissues.

## Discussion

There is a critical need to develop new therapeutic approaches for TNBC with residual tumors after neoadjuvant chemotherapy. In this study, we demonstrate that the inhibition of both Wnt/β-catenin and RAS/ERK pathways, especially via degradation of β-catenin and RAS, could be an effective therapeutic strategy for treating TNBC patients. This approach could be especially beneficial for TNBC treatment, since EGFR, which is overexpressed in approximately 50% of TNBCs, can be transcriptionally suppressed through Wnt/β-catenin pathway inhibition. The pathological significance of our small-molecule approach, which simultaneously reduced RAS, β-catenin, and EGFR levels, was supported by their correlated overexpression in TNBC patient tumor tissues compared with the adjacent normal mammary glands in our survey using tissue microarray.

We demonstrated that the mechanism for RAS/ERK pathway regulation by Wnt/β-catenin signaling, and the co-regulation of β-catenin and RAS stability in CRC^27–30^ is also conserved in TNBC, as confirmed by in vitro experiments that showed the effects of Wnt3a treatment in MCF10A.

The suppressive effects of KYA1797K on proliferation and transformation via β-catenin and RAS degradation and the transcriptional inhibition of *EGFR* were shown in primary and established TNBC cells. This transcriptional inhibition of *EGFR* by KYA1797K may be caused via suppression of Wnt/β-catenin signaling, as indicated by the finding that *EGFR* is a transcription target of the pathway^14^.

In addition, the inhibition of mobility and invasion of TNBC cells by KYA1797K and the reduction in the corresponding biomarkers indicated that inhibition of both pathways provides a potential approach to overcome metastasis, which frequently occurs in TNBC patients^13^.

Regarding the causes of recurrence and metastasis after chemotherapy in TNBC, CSCs have been reported as a major triggering factor^31^. The inhibition of 3D-cultured organoids or spheroids derived from TNBC patient cells or established TNBC cell lines after KYA1797K treatment indicates its role in CSC suppression. Finally, we also confirmed the effectiveness of KYA1797K by the growth reduction of transplanted tumors in mice that were established from MDA-MB-468 or TNBC patient tissues. The clinical significance of β-catenin and RAS destabilization, and ultimately EGFR transcriptional suppression, was confirmed by the PDX experiment using residual tumors from TNBC patients after neoadjuvant chemotherapy. Although the kinetics and degree of growth reduction after KYA1797K treatment varied between PDX-1 and PDX-2 (Supplementary Fig. 7), this may be attributed to the heterogeneity associated with characteristics of the patient tumors.

Overall, we found that β-catenin, RAS, and EGFR proteins are correlatively overexpressed in human TNBC. We further demonstrated that KYA1797K lowered the expression of these three proteins, which suppressed the growth of TNBC cells in both in vitro and in vivo systems, as using PDX and patient primary cells. Our small-molecule approach, which suppressed both Wnt/β-catenin and RAS-ERK pathways with a subsequent reduction in EGFR expression, could be a potential therapeutic strategy for TNBC.

## Supplementary information

Supplementary information
