# Supplementary material for: Destabilization of β-catenin and RAS by targeting the Wnt/β-catenin pathway as a potential treatment for triple-negative breast cancer
Source: Exp Mol Med. 2020 May 26;52(5):832–42. doi: 10.1038/s12276-020-0440-y (PMC7272395; doi:10.1038/s12276-020-0440-y)
Supplement: Supplementary file 1 — Supplementary information [file 12276_2020_440_MOESM1_ESM.docx]

**Supplementary Information
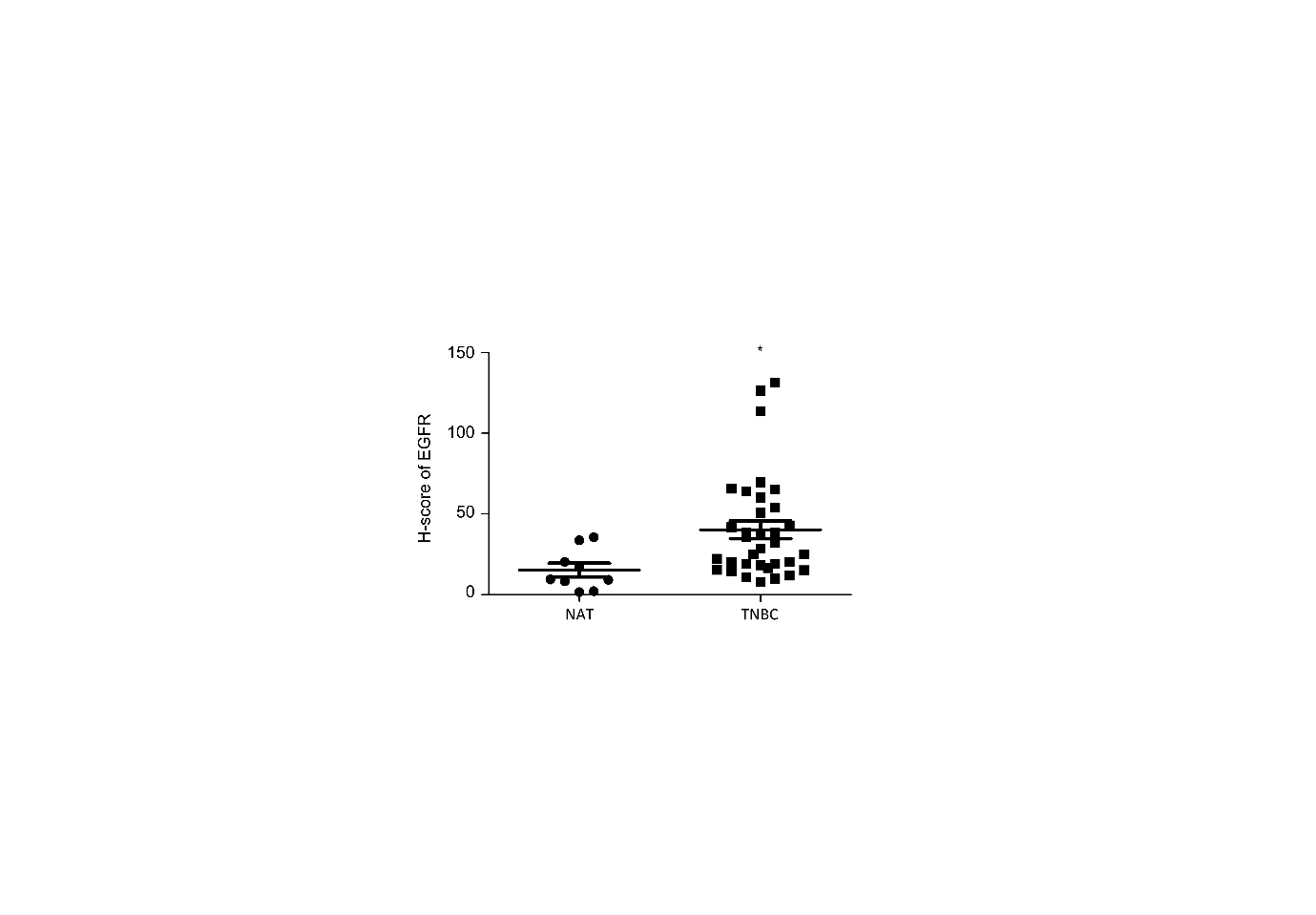
**

**Supplementary Fig. 1. Expression of EGFR in normal-adjustment tissues (NAT) and TNBC patient tumor tissues.** Quantification of the histoscore (H-score) of membranous EGFR (*p* < 0.05) in NAT and TNBC tumor tissues using Graph Pad Prism 5 (*t*-test with two-tailed *p*-value).
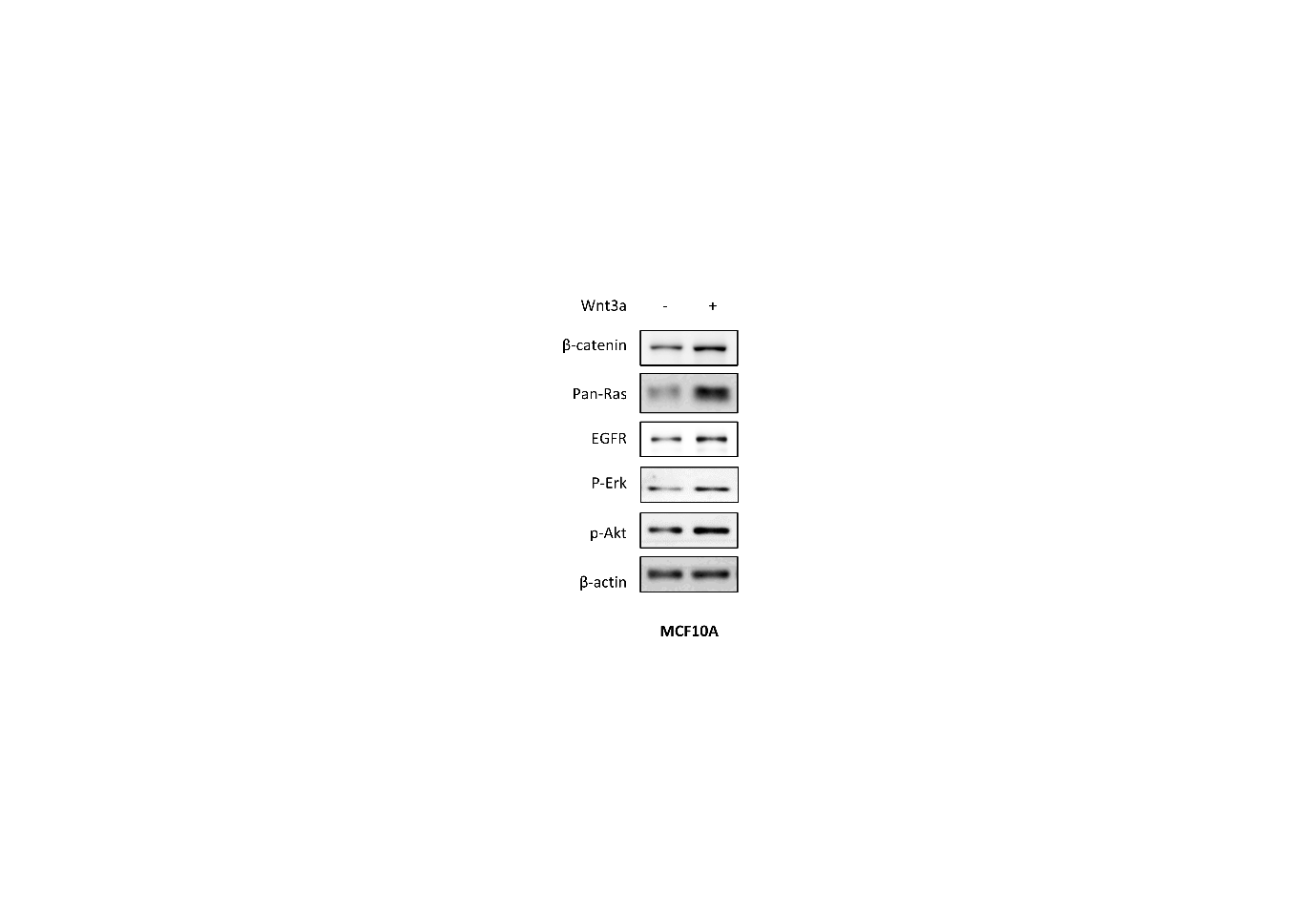


**Supplementary Fig. 2. Effects of Wnt3a on Wnt/β-catenin and RAS/ERK pathway components in TNBC cell line.** MCF10A cells were treated with PBS or 50 ng/mL of recombinant Wnt3a for 24 h. Whole cell lysates (WCL) were subjected to immnoblotting (IB) analyses with indicated antibodies.


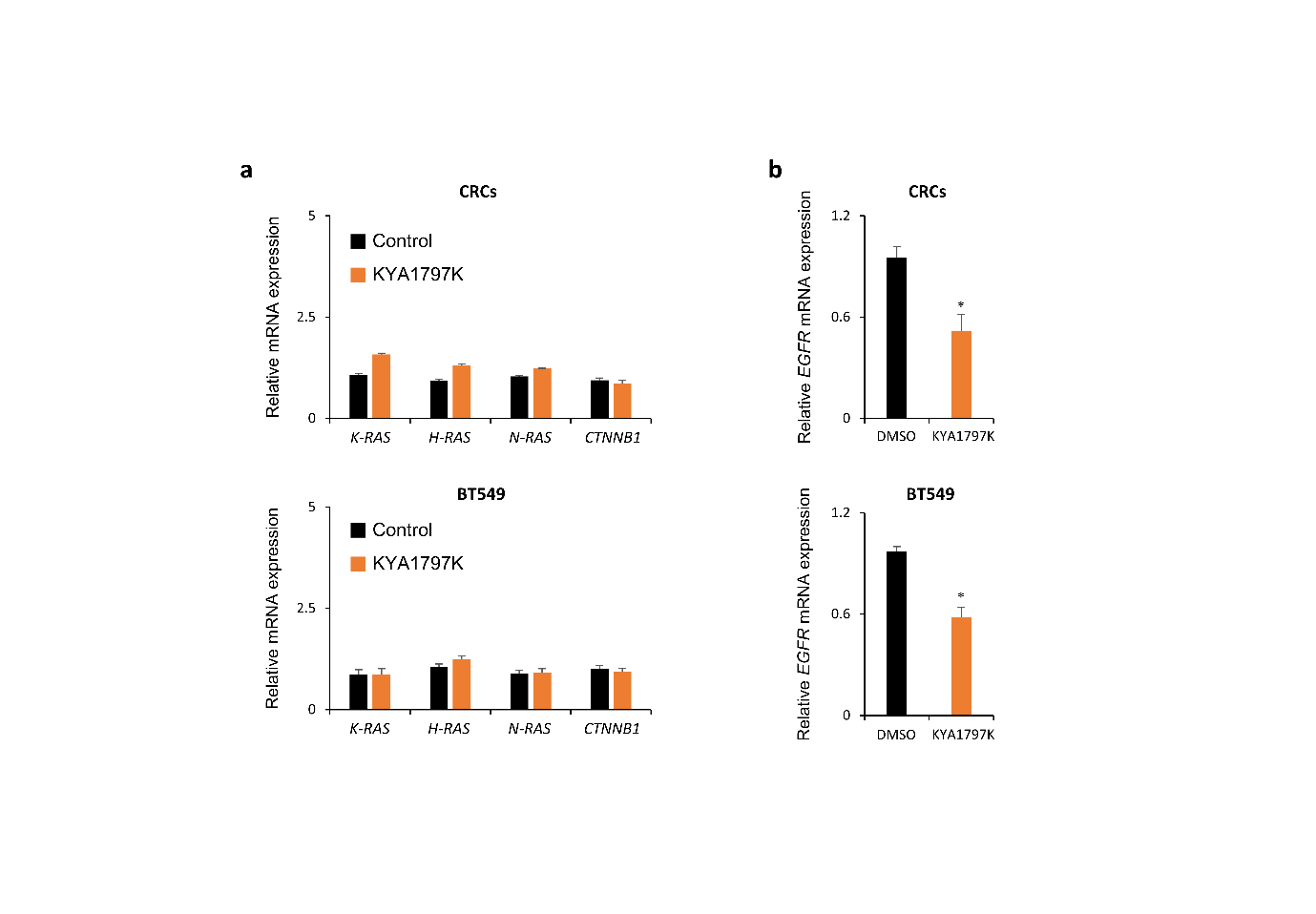


**Supplementary Fig. 3 Effect of KYA1797K on mRNA expression in TNBC cells.** (a, b) Real-time quantitative polymerase chain reaction (qPCR) assays were used to determine the mRNA expressions of the *K-*, *H-*, *N-RAS*, *CTNNB1*, and *EGFR* in CRCs and BT549 cells treated with DMSO or KYA1797K. Data values represent mean ± SEM. **P* < 0.05, ***P* < 0.005, ****P* < 0.0005


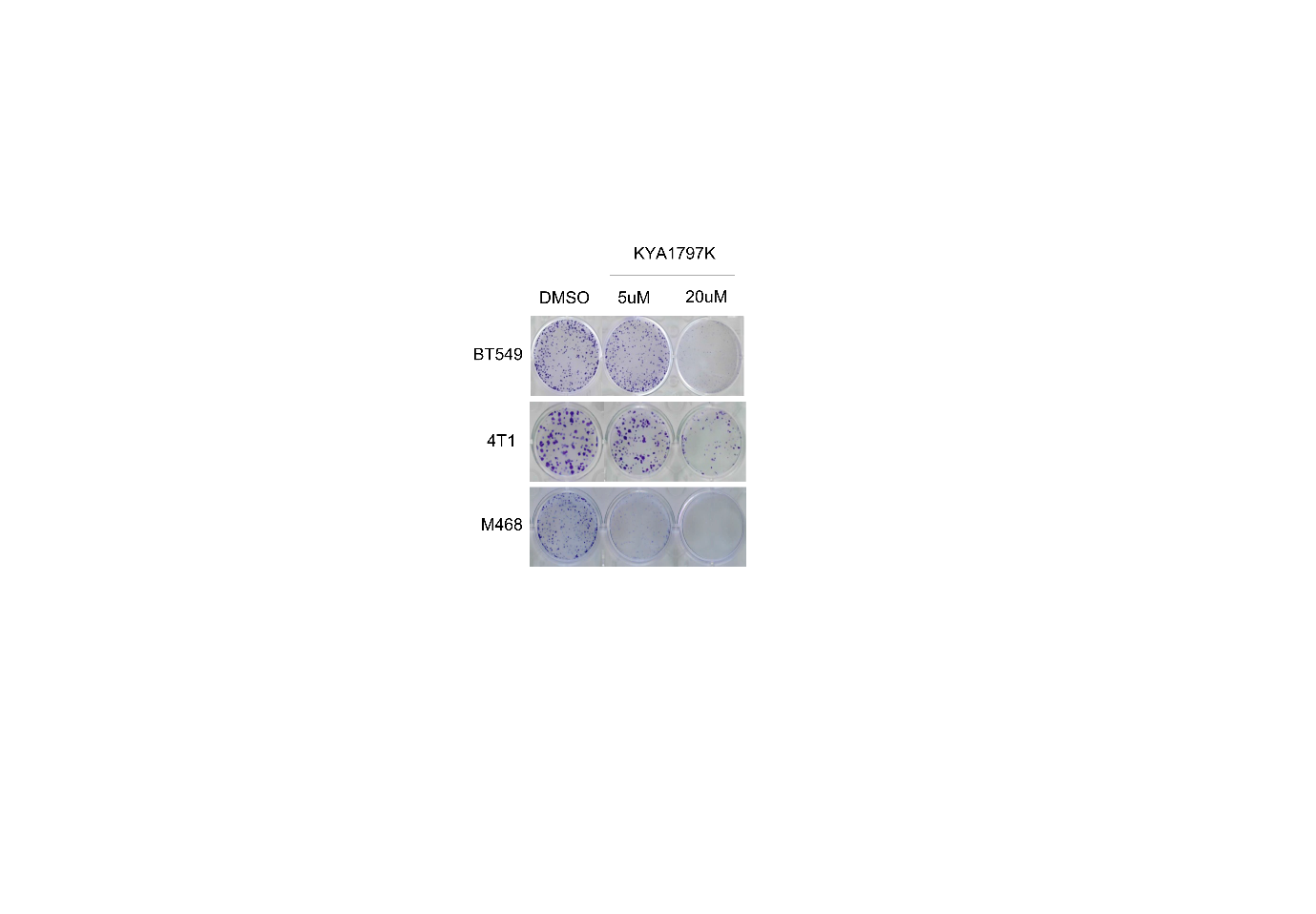


**Supplementary Fig. 4. Effect of KYA1797K on transforming ability of TNBCs.** The representative images of colonies of TNBC cells corresponding to Figure 2. MDA-MB-468 cells were treated with KYA1797K for 14 days as described in Methods.


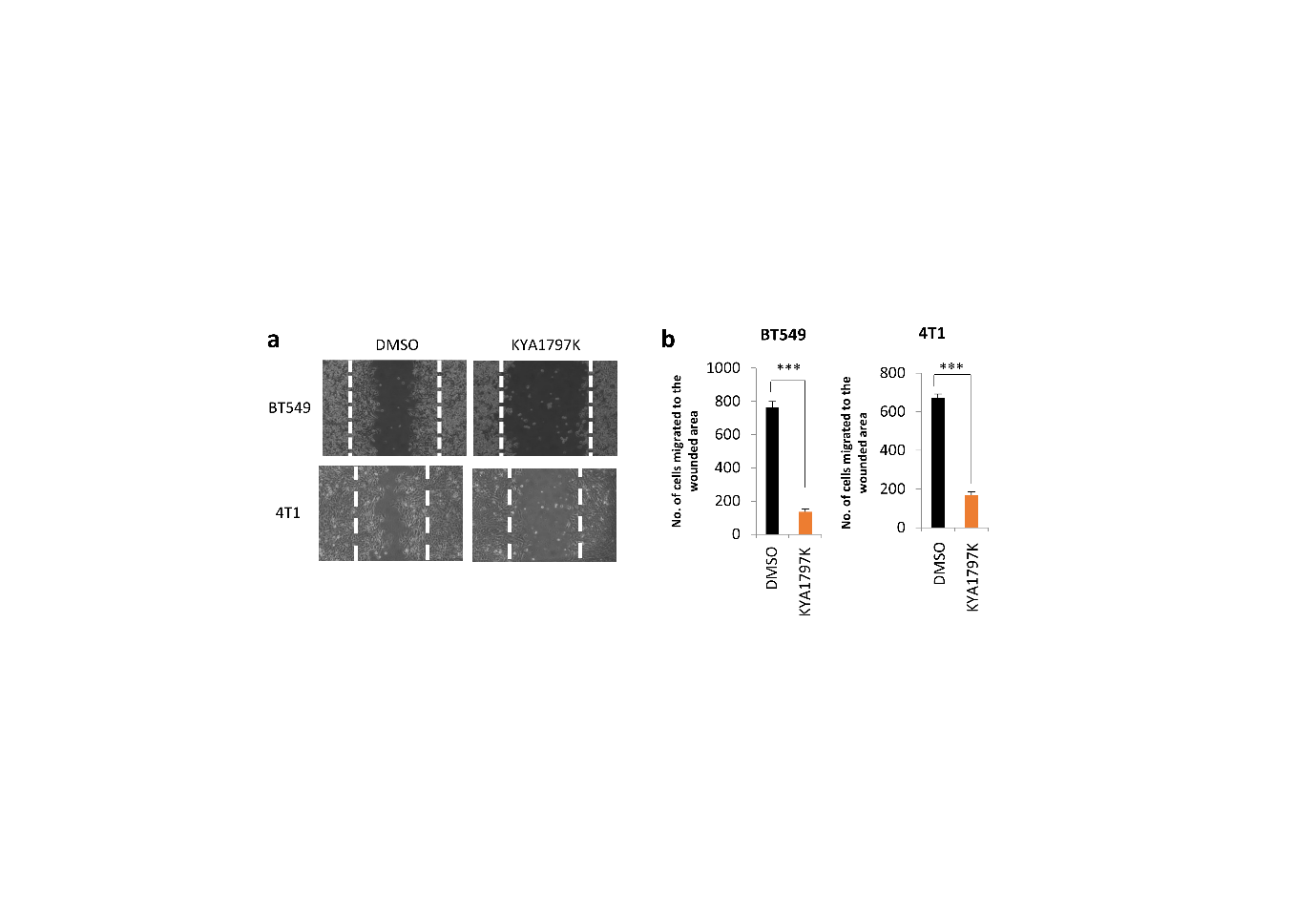


**Supplementary Fig. 5. Effect of KYA1797K in migration of TNBC cells.** BT549 and 4T1 cells were seeded in 12-well plate for migration assay. Confluent cells were scratched, and treated with KYA1797K for 24 h. (a, b) Representative images of wound closure and quantification of migratory cells are provided. Data values represent mean ± SEM. **P* < 0.05, ***P* < 0.005, ****P* < 0.0005


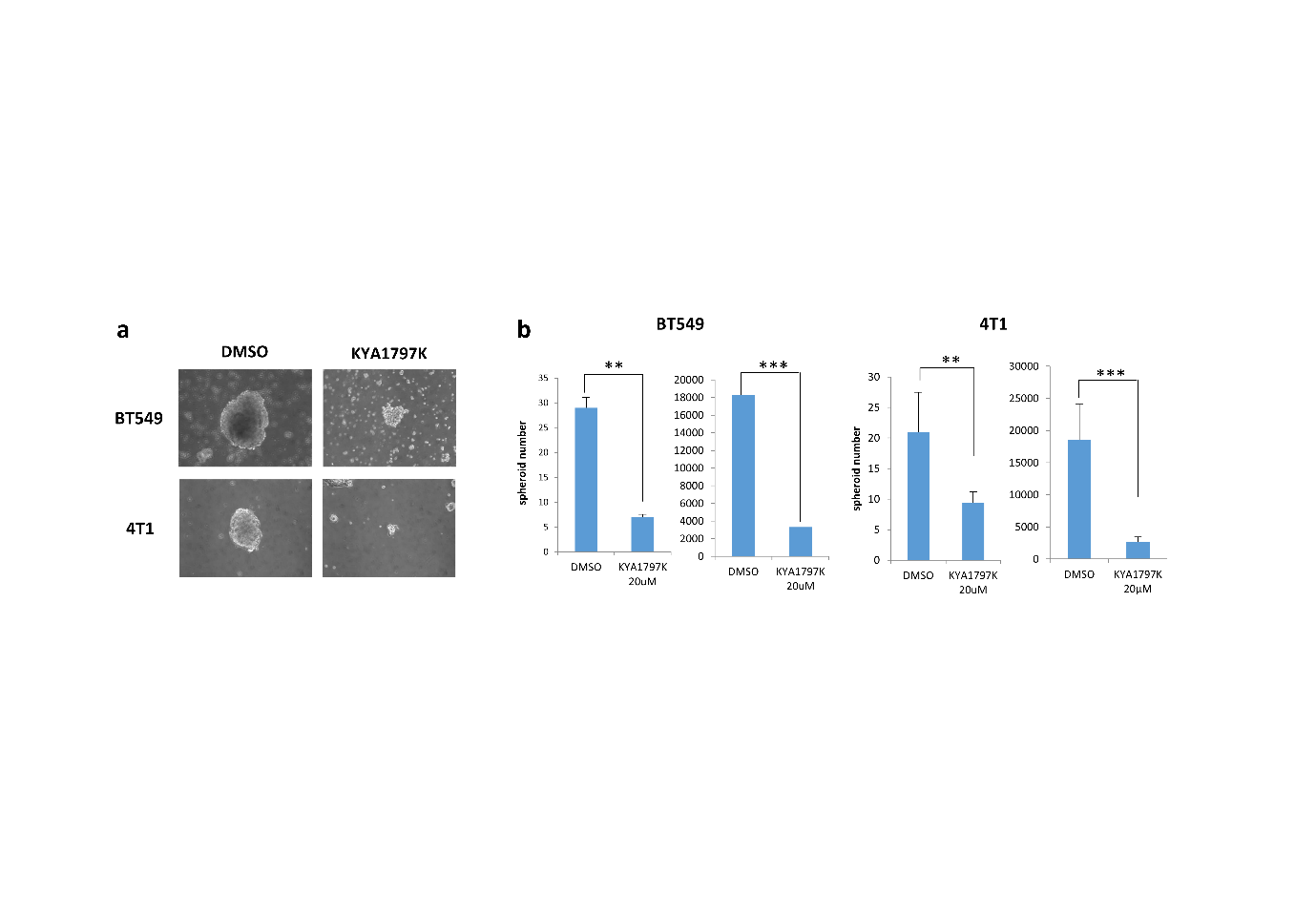


**Supplementary Fig. 6. Suppression of stemness of TNBC cells by KYA1797K.** BT549 and 4T1 cells were seeded at a density of 1 × 10^4^ (*n* = 3) cells/plate in 90 mm petri dishes to allow spheroid (SP) formation. Spheroids were treated with KYA1797K from day 3 of seeding and medium was changed every 2 days. (a) Representative images were captured by Nikon TE2000U at the end of experiment. (b) The number and size of spheroids were quantified and two-sided Student’s *t*-test was used to determine statistical significance. Data values represent mean ± SEM. **P* < 0.05, ***P* < 0.005, ****P* < 0.0005


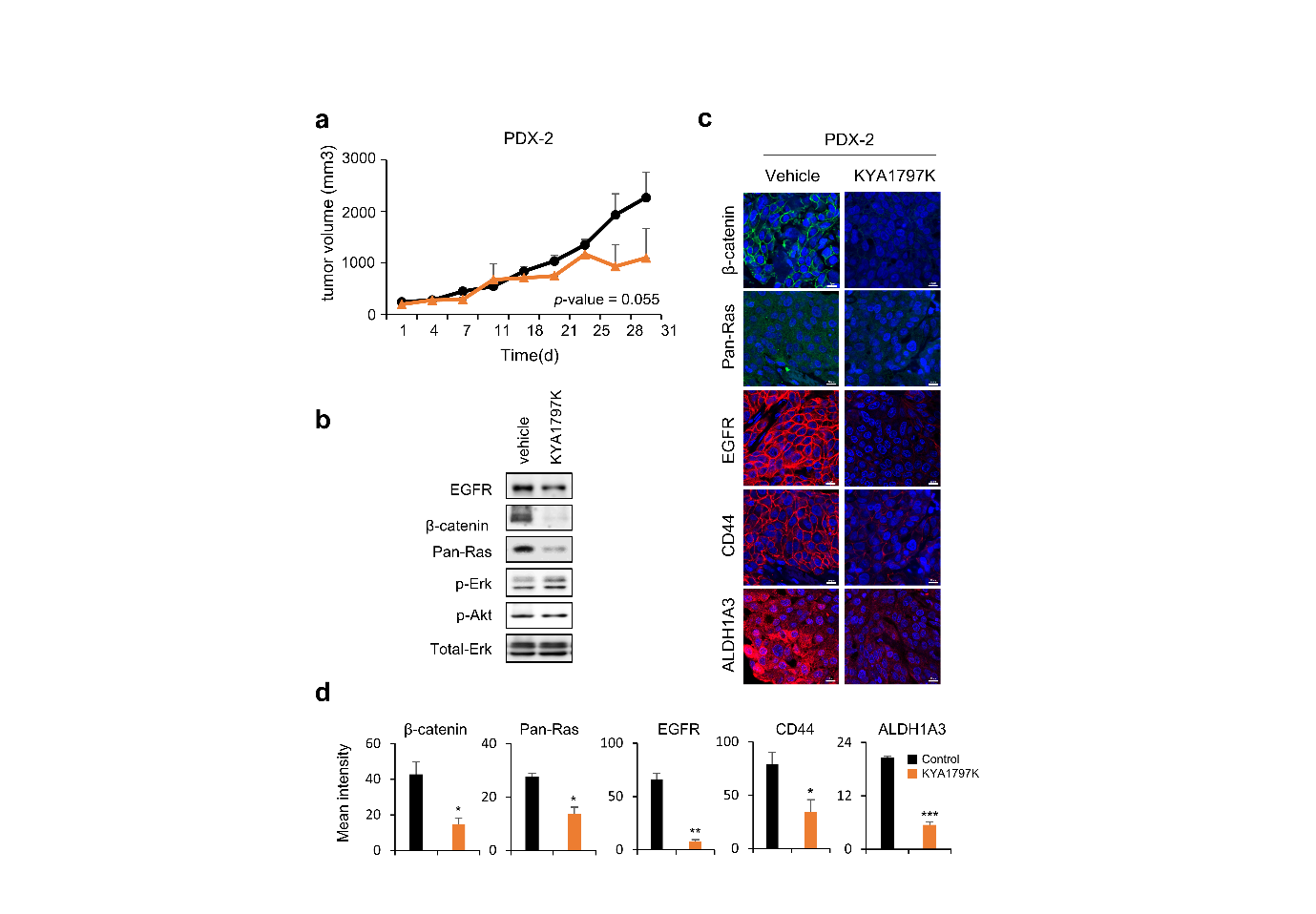


**Supplementary Fig. 7. Inhibitory effects of KYA1797K on PDX tumors derived from a TNBC patients.** Mice bearing TNBC tumors, which were established from the patients that showed resistance to adjuvant chemotherapy, were treated with vehicle or KYA1797K. (a) Once the tumor sizes reached volumes of 150–200 mm3, tumor volumes were measured for 31 days (PDX-2). (b) IB analyses using whole cell lysates (WCLs) of PDX-2 tumors for indicated antibodies. (c, d) IHC analyses were performed using indicated antibodies on formalin-fixed 4 μm paraffin sections of the PDX-2 tumors. Mean fluorescence intensity for each marker was quantified by ZEN microscope software (Carl Zeiss Microscopy). Data values represent mean ± SEM. **P* < 0.05, ***P* < 0.005, ****P* < 0.0005

**
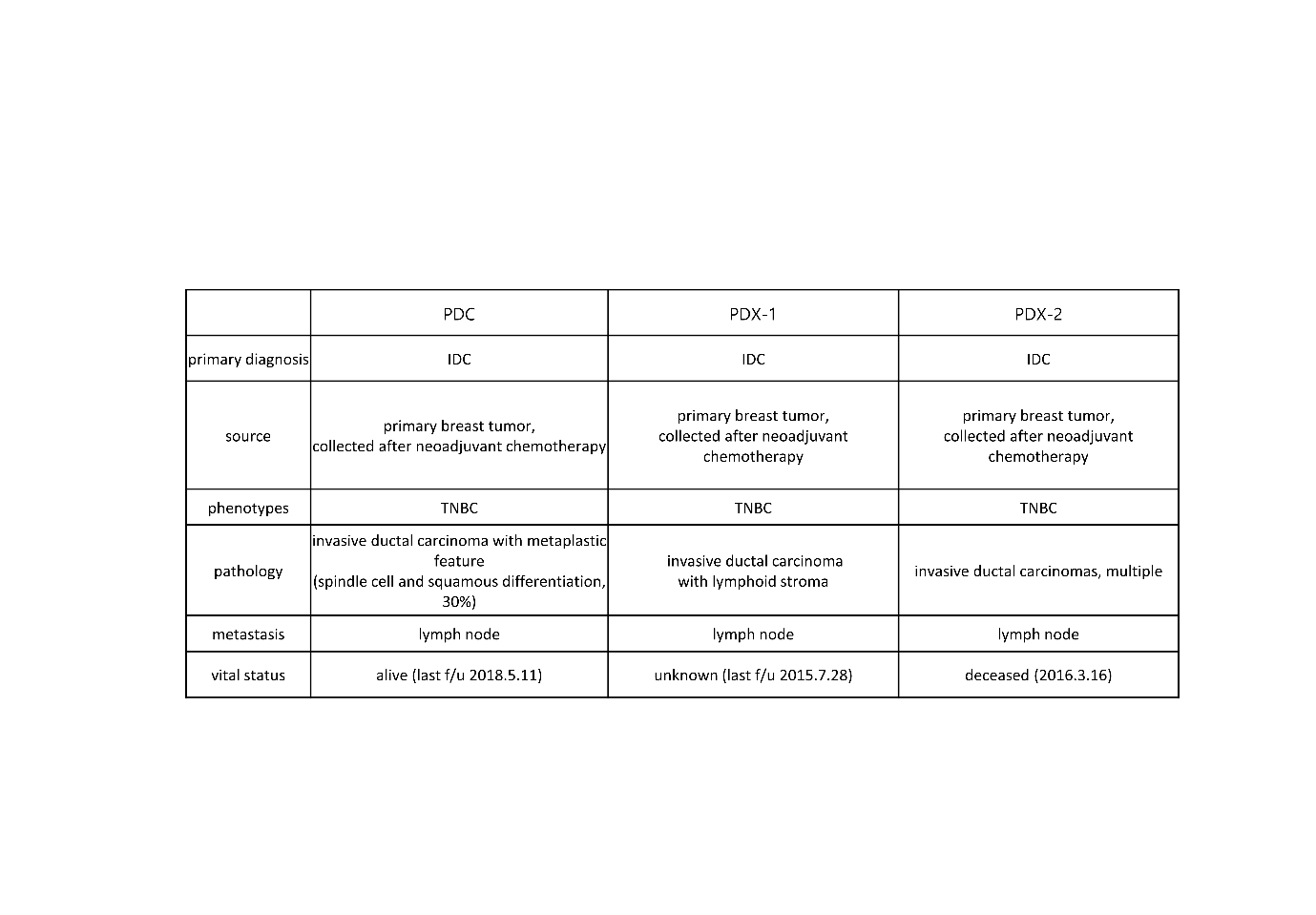
**

**Supplementary Table 1. Clinical information of TNBC patients**
